# Socio-Demographic Correlates of Cycling to School among 12- to 15-Year Olds in Southern Germany

**DOI:** 10.3390/ijerph17249269

**Published:** 2020-12-11

**Authors:** Dorothea M. I. Schönbach, Catherina Brindley, Anne K Reimers, Adilson Marques, Yolanda Demetriou

**Affiliations:** 1 Department of Sport and Health Sciences, Technical University of Munich, 80992 Munich, Germany; yolanda.demetriou@tum.de; 2 Department of Natural and Sociological Sciences, Heidelberg University of Education, 69120 Heidelberg, Germany; brindley@ph-heidelberg.de; 3 Department of Sport Science and Sport, Friedrich-Alexander-University Erlangen-Nuremberg, 91058 Erlangen, Germany; anne.reimers@fau.de; 4 CIPER, Faculty of Human Kinetics, University of Lisbon, 1499-002 Lisbon, Portugal; amarques@fmh.ulisboa.pt

**Keywords:** associations, girls, boys, mothers, fathers, bicycle, active commuting to school

## Abstract

Depending on the region and urbanization level, the rate of cycling to school in Germany varies largely. The influence of distance from home to school, educational level, the school’s region, and parents’ socio-demographic characteristics on cycling to secondary school in Germany is unclear. Therefore, this study analyzed students’ and parents’ socio-demographic correlates of cycling to school, including separate analyses by gender, among 12- to 15-year-olds attending different (sub)urban schools in Southern Germany. In 2019, 121 students (girls: 40.5%, boys: 59.5%) aged 13.1 ± 0.9 and 42 parents (mothers: 81%, fathers: 19%) aged 47.8 ± 5.5 participated. Students completed a self-report questionnaire; parents completed a self- and proxy-report questionnaire. In total, between 61.7% and 67.5% of students sometimes cycled to school. Binary logistic regressions revealed that being a girl, increasing age, attending an intermediate educational level combined with a suburban school region (small or medium-sized town), increasing distance from home to school, and having parents who did not cycle to work led to declining odds of cycling to school. Many 12- to 15-year-olds sometimes cycled to school in (sub)urban school regions in Southern Germany. As several socio-demographic characteristics correlated with cycling to school, this should be considered when developing a future school-based bicycle intervention.

## 1. Introduction

Physical activity (PA) has positive impacts on physical, psychosocial, and cognitive health in children and adolescents aged 5 to 17 [1]. Nevertheless, only 26% of children and adolescents (girls: 22.4%; boys: 29.4%) aged 3 to 17 in Germany achieve the PA guidelines of the World Health Organization [2]. Children and adolescents aged 5 to 15 who cycle to school have the highest chance to achieve these guidelines weekly (cyclists: 36%; walkers: 25%, neither cyclists nor walkers: 22%) [3]. Additionally, cardiorespiratory [4,5,6] as well as cardiovascular fitness [7] are positively associated with cycling to school in children and adolescents, possibly due to a higher intensity of PA [7]. A higher PA intensity is associated with more solid health benefits in children and adolescents [1]. Therefore, cycling to school might have better health-related outcomes than other means of transportation.

Previous studies conducted in Germany between 2003 and 2019 reported different rates of cycling to school, especially among adolescents. In nationwide representative samples of 11- to 17-year olds, the rate of cycling to school was consistently low but slightly increased [8,9]. Between 2003 and 2006, 22.2% within this age group (girls: 20.6%, boys: 23.8%) usually cycled to school [8], while 21.5% of girls and 25.2% of boys usually cycled to school between 2014 and 2017 [9]. In a regionally representative study conducted in a city located in Northern Germany, 50% of adolescents aged 14 typically cycled to school between 2004 and 2005 [10]. Another regionally representative study conducted in a city located in Southern Germany in 2005 showed that 8% of students from grades 3 to 13 cycled to school daily [11]. In our recent non-representative study from 2019, conducted in a small town, a medium-sized town and a city located in Southern Germany, 44.4% of girls and 72.9% of boys aged 12 to 15 sometimes cycled to school [12]. Following this, the rate of cycling to school among children and adolescents in Germany varies largely depending on the context, i.e., sampled region(s) and the level of urbanization. According to the Global Matrix 3.0, Germany was graded with C– based on reports that ca. 40% of children and adolescents use active modes to commute to school [13].

We chose the age range 12 to 15 as a study conducted in Finland between 1980 and 2007 found that the rate of active commuting to school (ACTS), including both walking and cycling, decreased sharply between the ages of 12 and 15 [14], suggesting that this age range might be a high-risk population. Similar findings were reported for cycling to school in a study conducted in Colombia where more children up to 12 years cycled to school compared with adolescents aged 13 and older [15]. However, we reported very high rates of cycling to school among 12- to 15-year olds in our recent study [12], suggesting that the contexts in which cycling to school occurs also vary by countries.

In the model of children’s active travel (M-CAT), characteristics of the child (e.g., gender, age, or school attended) and its parents (e.g., employment or socioeconomic status (SES)) impact the decision-making process to actively travel to school [16]. Furthermore, M-CAT highlights the influence of those characteristics (e.g., gender) on the perceptions of the child and its parents, which affects the ultimate decision of the child to actively travel to school while taking into account how parents had decided on allowance or restriction [16]. With increasing age of the child, the influence of its parents’ decision decreases [16]. In particular for cycling to school, previous studies have identified age [3,9,15,17], gender [3,8,11,15,17,18], migration background [8,19], weight status [19,20,21], distance from home to school [11,22,23], residential area [8], SES [9,24], and child’s [11]/parents’ educational level [15] as socio-demographic correlates. However, the contributing role of distance from home to school among secondary school students has only been examined for one city in Germany [11]. Moreover, the influence of the child’s educational level on cycling to school among secondary school students has only been examined for one city in Germany [11] and never in other countries. The role of parents’ socio-demographic characteristics is generally unclear, not only in Germany. Additionally, the school’s region has never been studied in previous research up to now, neither in Germany nor in other countries.

Thus, this study aimed to determine the correlations of students’ and parents’ socio-demographic characteristics with habits of cycling to school among 12- to 15-year olds attending different educational levels of schools located in different (sub)urban regions in Southern Germany and to analyze correlates concerning the gender of students as well as parents. When identifying those correlates of cycling to school, researchers can address them in future school-based bicycle interventions.

## 2. Methods

### 2.1. Study Design

We analyzed data from 121 out of 154 students (49 girls, 72 boys) aged 13.1 ± 0.9 (see Table A1) and 42 parents (34 mothers, 8 fathers) aged 47.8 ± 5.5 (see Table A3) from a study conducted in Germany in 2019 aiming to understand what is needed to cycle to school daily according to students, parents, and teachers [12]. Data was collected at three secondary schools, each including two classes of seventh and/or eighth graders aged 12 to 15, with two different educational levels (intermediate = two schools, high = one school) located in urban (one school in a city with 1.5 m inhabitants) and suburban (one school in a small town with 13,000 inhabitants and one school in a medium-sized town with 21,000 inhabitants) regions in Southern Germany. The medium-sized town and city were rated as (in)sufficient in a ranking for the satisfaction of cyclists in Germany [25], whereas no scientific evaluation on bikeability is available for the small town. However, the bicycle-friendliness for students in the small town appears to be rather low as there are no bicycle lanes.

### 2.2. Data Collection

The study comprised a sample of students and a sample of their parents. Both questionnaires were delivered independently of each other. Parents received an information letter and provided signed consent forms for themselves and on behalf of their child before the beginning of data collection. Prior to data collection, students and parents were instructed to produce a five-digit ID-code themselves, respectively, which ensured anonymity. Students completed a printed or online version of the questionnaire via the program Survalyzer (Survalyzer AG, Zurich, Switzerland) [26] at school, supervised by at least one trained researcher (D.M.I.S./C.B.). Parents completed an online version of the questionnaire via Survalyzer at home.

### 2.3. Measures

#### 2.3.1. Socio-Demographic Characteristics and Cycling to School in the Sample of Students

Based on self- and proxy-reported correlates of cycling to school in children and adolescents found in previous studies [3,8,9,11,15,17,18,22,23] as well as in a child and parental questionnaire on specific determinants of cycling to school [27], students were asked to provide the following socio-demographic characteristics in a self-report questionnaire: (a) age; (b) gender; (c) educational level; (d) region of the school (urban/suburban, number of inhabitants); (e) bicycle ownership; (f) ability to cycle; and (g) habit, frequency, and distance of cycling to school.

#### 2.3.2. Socio-Demographic Characteristics and Cycling to School in the Sample of Parents

Due to separate data collections in students and their parents, students could not have been matched to their parents (i.e., data could not have been merged). This is why parents were asked similar questions to provide their child’s socio-demographic characteristics (proxy-report) and their own socio-demographic characteristics (self-report), based on previous studies [28,29,30,31], in a questionnaire: (a) parents’/child’s age; (b) parents’/child’s gender; (c) child’s educational level; (d) region of child’s school (urban/suburban, number of inhabitants); (e) parents’/child’s bicycle ownership; (f) parents’/child’s ability to cycle; (g) parents’/child’s habit, frequency, and distance of cycling to school/work; (h) employment status; and (i) number of working days a week.

#### 2.3.3. Distance from Home to School

Previous research has shown that the actual cycling route is not longer than the shortest route [32]. Furthermore, the shortest route is easier to estimate with Google Maps (Google LLC, Mountain View, USA), which objectively quantifies the distance from home to school. Following this, distance from home to school was estimated by participants for the shortest rather than the actual route by foot using Google Maps.

### 2.4. Statistical Analysis

All analyses were performed using the program IBM SPSS Statistics 25 (IBM Corporation, Armonk, USA) [33]. Only female and male participants who completed data collection on socio-demographic characteristics were included in this analysis. Binary logistic regressions were conducted, for which a minimum sample size of 50 is recommended [34]. Separate analyses were performed to determine associations between the habits of cycling to school among 12- to 15-year-olds (as a dependent variable) and each of the independent variables collected in the sample of students (self-reported socio-demographic characteristics: age, gender, educational level/school’s region, number of inhabitants, and distance from home to school) as well as parents (proxy-reported socio-demographic characteristics for their own child: age, gender, educational level/school’s region, number of inhabitants, and distance from home to school; self-reported socio-demographic characteristics: age, gender, employment status, number of working days a week, and habit/frequency/distance of cycling to work). Additionally, separate gender analyses were performed for the sample of students (i.e., girls and boys) and parents (i.e., mothers). No separate gender analysis for fathers was performed as the number of participants was too small (*n* = 8). Predicted probability in all analyses is of giving a negative answer to the question: “Do you cycle to school sometimes?”. The reference group was set based on the favored population according to the current state of the literature.

## 3. Results

### 3.1. Students’ Socio-Demographic Characteristics as Correlates of their Cycling to School Habits

In total, 95% of students owned a bicycle and 61.7% of students sometimes cycled to school, of which 35.7% cycled to school daily (see Table A1). On average, students generally cycled to school on 2.3 ± 2.0 days a week.

The results of the binary logistic regressions for students’ habits of cycling to school showed that students (girls and boys) attending an intermediate educational level combined with a suburban school region (*p* = 0.035; OR = 2.5 [CI 95 for OR: 1.1, 5.8]) and girls (*p* = 0.003; OR = 3.4 [CI 95 for OR: 1.5, 7.4]) were less likely to cycle to school (see Table A2). Moreover, cycling to school among students (girls and boys) became less likely with increasing age (*p* = 0.002; OR = 2.1 [CI 95 for OR: 1.3, 3.3]) and when attending a school located in a small town (*p* = 0.010; OR = 3.5 [CI 95 for OR: 1.4, 8.9]). Both associations were mainly due to girls according to the results of the separate gender analysis as no correlates were found in the separate gender analysis of boys.

### 3.2. Parents’ and their Child’s Socio-Demographic Characteristics as Correlates of their Child’s Cycling to School Habit

All parents reported that their child owned a bicycle and 67.5% of parents indicated that their child sometimes cycled to school, of which 63% cycled to school daily according to parents (see Table A3). On average, parents stated that children generally cycled to school on 2.7 ± 2.3 days a week.

The results of the binary logistic regressions for the child’s habit of cycling to school reported by parents (mothers and fathers) showed that cycling to school became less likely when the child’s parent did not cycle to work (*p* = 0.043; OR = 5.9 [CI 95 for OR: 1.1, 32.9]) (see Table A4). Moreover, proxy-reports of parents (mothers and fathers) revealed that children were less likely to cycle to school when attending an intermediate educational level combined with a suburban school region (*p* = 0.010; OR = 9.4 [CI 95 for OR: 1.7, 51.0]), attending a school located in a medium-sized town (*p* = 0.008; OR = 10.6 [CI 95 for OR: 1.9, 60.2]), and living further away from school (*p* = 0.006; OR = 1.4 [CI 95 for OR: 1.1, 1.8]). These three associations were also found in the separate gender analysis of mothers’ proxy reports.

## 4. Discussion

The purposes of this study were to determine the correlations of students’ and parents’ socio-demographic characteristics with 12- to 15-year-olds’ habits of cycling to school, who attended different educational levels of schools located in different (sub)urban regions in Southern Germany, and to consider gender in the analyses.

More than half of the students sometimes cycled and one-third to two-thirds cycled daily to school in this study, which are the highest rates compared to all other studies reporting cycling to school rates in Germany [8,9,10,11]. As correlates of cycling to school, attending an intermediate educational level in combination with a suburban region of the school led to a lower likelihood to be engaged in cycling to school. Girls were less likely to cycle to school than boys. Mainly due to girls, attending a school located in a small town and increasing age were also identified as inhibitive factors. Living further away from school as well as attending a school located in a medium-sized town and having parents not using a bicycle to commute to work were negatively associated with 12- to 15-year-olds cycling to school habits.

### 4.1. Rate and Correlates of Cycling to School

The high rate of cycling to school in our samples might be explained by the sizes and characteristics of the included municipalities (suburban = small town and medium-sized town, urban = city) and the gender ratio in favor of boys (59.5% boys participated and parents referred to 64.3% sons). A rural region, which was not included in our study, was identified as the strongest barrier of cycling to school in previous research [8], whereas being a boy was an advantage [3,8,11,15,17,18]. Although previous research reported that a lower urbanization level (i.e., a medium-sized town compared to a city) was positively associated with cycling to school [8], we found the opposite relationship in parents’ proxy-reports. This contrary finding confirmed the dependency of the context, i.e., sampled municipalities. Concerning gender differences, girls mentioned an additional gender-specific need (i.e., social behavior in road traffic) in order to cycle to school daily compared with boys, who did not mention this need, in our recent study including the same sample [12]. This could explain the high rates of boys cycling to school if the specific girls’ need is not sufficiently addressed. Additionally, we found a first indication in our previous systematic review that poorer health-related fitness among girls, possibly due to engaging less in PA overall, could be a barrier to uptake cycling with its moderate-to-vigorous intensity [35].

It remained unclear which of the two factors, i.e., the educational level of students or the school’s region, or a combination of both were associated with a lower probability of cycling to school. The reason for this is that there was no variance between the combination of both factors in the present study (i.e., only one school with a high educational level located in the urban region and two schools with intermediate education levels located in suburban regions). In previous research, it has been suggested that regions with a lower urbanization level are characterized by a lower school density, which can lead to a longer distance from home to school [8] and this lowers the chance of cycling to school [11,23]. Concerning the influence of educational levels in students, a previous assumption that bicycle ownership could be a limiting factor [9] is not reasonable in our study as almost all students owned a bicycle in line with the official report of a German Federal Ministry [36]. However, it remains unclear whether these bicycles are roadworthy, usable, and suitable. We rather support the idea mentioned in a previous study [24] that factors not considered in our analyses (e.g., the social norm among peers [37]) might explain this finding.

Increasing age, especially in girls, was associated with a declining habit of cycling to school, which is in line with the current state of research [15] reporting that the stability of PA in transitional phases (e.g., from childhood to adolescence) was found to be lower due to growth and life-changing events [38].

Finally, parents’ habits of cycling to work appeared to serve as supportive role modeling [28,29,30], which could be an explanation for the association with children’s cycling to school odds. However, mothers but not students acknowledged the role of parents in our recent study [12], suggesting that social norms play an unconscious role [37]. In contrast to previous research targeting ACTS [28,30], we did not find a relationship between mothers’ habits of cycling to work and children’s cycling to school habits. As no gender analysis could be made for fathers, it remains unclear if the fathers’ gender matters in this finding [28,30].

### 4.2. Strengths and Limitations

The major strengths of this study are to focus on the high-risk group of 12- to 15-year-olds in terms of cycling to school and to identify inhibitive or supportive socio-demographic characteristics of students as well as parents, including separate analyses for gender. Moreover, our study is the first in Germany that considered distance from home to school and educational levels in secondary school students who cycle to school in more than one city and state. In general, the influence of the school’s region was studied for the first time. Compared to the high response rate of students at schools, the number of participating parents at home was relatively low. The conclusions drawn from our findings are limited due to the small, non-representative sample size, the restriction to (sub)urban regions in Southern Germany, and selective educational levels (i.e., intermediate and high). Additionally, it must be acknowledged that the reliability of estimated effect sizes is uncertain in some findings. Also, this study did not provide insights into correlates associated with fathers’ socio-demographic characteristics. Furthermore, information about SES and residential area was not directly assessed. Migration background, weight status, and parents’ educational level were not considered.

## 5. Conclusions

Although conclusions can only be drawn with caution, our findings give new insights into habits of cycling to school and its influencing factors in Germany. This study indicated that approximately every second student aged 12 to 15 sometimes cycled to school in Southern Germany. We observed that several socio-demographic characteristics of students and parents, i.e., gender, age, educational level/school’s region (urban/suburban, number of inhabitants), distance from home to school, and parents’ habits of cycling to work, were correlated with habits of cycling to school. These findings suggest that it is essential to address the gender-specific need of girls, stabilize habits of cycling to school in the transition from childhood to adolescence, establish supportive social norms, and involve parents as role models in future school-based bicycle interventions. Concerning the barrier “distance from home to school”, the active part on the way to school could be shortened by splitting the way into active and passive parts (if necessary) as all three schools are closely located to public transport facilities. Furthermore, we suggest inviting parents to school for data collection to ensure a high response rate [12]. Finally, more research regarding the (gender) influence of parents’ socio-demographic characteristics on children’s cycling to school habits is warranted as there are many other possible socio-demographic characteristics in parents that have not been analyzed up to now, e.g., marital status [16], number of children [16], or car availability [39].

## Abbreviations

ACTS = active commuting to school; ca. = circa; CI = confidence interval; e.g. = for example; i.e. = that is; m = million; M-CAT = model of children’s active travel; *n* = sample size; OR = odds ratio; *p* = probability value; PA = physical activity, SES = socioeconomic status.

## Appendix A

**Table A1:** Socio-Demographic Characteristics based on the Sample of Students.

Socio-Demographic Characteristics	Girls (*n* = 49)	Boys (*n* = 72)	Total (*N* = 121)
Age (years in M ± SD)	13.1 ± 0.9	13.1 ± 0.9	13.1 ± 0.9
Educational level/school’s region			
(a) high/urban	13 (26.5%)	29 (40.3%)	42 (34.7%)
(b) intermediate/suburban	36 (73.5%)	43 (59.7%)	79 (65.3%)
Number of inhabitants			
(a) city	13 (26.5%)	29 (40.3%)	42 (34.7%)
(b) medium-sized town	15 (30.6%)	22 (30.6%)	37 (30.6%)
(c) small town	21 (42.9%)	21 (29.2%)	42 (34.7%)
Bicycle ownership			
(a) yes	43 (87.8%)	72 (100%)	115 (95.0%)
(b) no	6 (12.2%)	0 (0%)	6 (5.0%)
Ability to cycle			
(a) yes	49 (100%)	72 (100%)	121 (100%)
(b) no	0 (0%)	0 (0%)	0 (0%)
Cycling to school (habit)			
(a) yes	20 (44.4%)	51 (72.9%)	71 (61.7%)
(b) no	25 (55.6%)	19 (27.1%)	44 (38.3%)
Cycling to school (days/week in M ± SD)	1.6 ± 2.0	2.7 ± 2.0	2.3 ± 2.0
Cycling distance home/school (km in M ± SD)	3.3 ± 2.6	4.0 ± 3.1	3.7 ± 2.9

km = kilometer; M = means; SD = standard deviation.

**Table A2:** Binary Logistic Regressions of Socio-Demographic Characteristics and Habits of Cycling to School based on the Sample of Students.

Socio-Demographic Characteristics	Girls				Boys				Girls and Boys			
	*p*	OR	95% CI for OR		*p*	OR	95% CI for OR		*p*	OR	95% CI for OR	
			Lower	Upper			Lower	Upper			Lower	Upper
Gender									**0.003**	3.4	1.5	7.4
(a) girls												
(b) boys (ref.)												
Age (in years)	**0.002**	4.9	1.8	13.6	0.329	1.4	0.7	2.5	**0.002**	2.1	1.3	3.3
Educational level/school’s region												
(a) high/urban (ref.)												
(b) intermediate/suburban	0.078	3.5	0.9	14.1	0.382	1.6	0.5	5	**0.035**	2.5	1.1	5.8
Number of inhabitants												
(a) city (ref.)												
(b) medium-sized town	0.790	1.3	0.2	6.4	0.353	1.8	0.5	6.6	0.336	1.6	0.6	4.5
(c) small town	**0.012**	8	1.6	40.6	0.566	1.5	0.4	5.4	**0.01**	3.5	1.4	8.9
Cycling distance home/school (in km)	0.098	1.3	1	1.7	0.469	1.1	0.9	1.3	0.226	1.1	1	1.2

CI = confidence interval; km = kilometer; OR = odds ratio; *p* = probability value; ref. = reference value.

**Table A3:** Parents’ and their Child’s Socio-Demographic Characteristics based on the Sample of Parents.

Socio-Demographic Characteristics	Mothers (*n* = 34)	Fathers (*n* = 8)	Total (*N* = 42)
Age (years in M ± SD)	46.8 ± 5.1	52.1 ± 5.2	47.8 ± 5.5
Age of child (years in M ± SD)	12.6 ± 0.7	13.0 ± 0.8	12.7 ± 0.7
Gender of child			
(a) daughter	12 (35.3%)	3 (37.5%)	15 (35.7%)
(b) son	22 (64.7%)	5 (62.5%)	27 (64.3%)
Educational level/school’s region of child			
(a) high/urban	15 (44.1%)	4 (50.0%)	19 (45.2%)
(b) intermediate/suburban	19 (55.9%)	4 (50.0%)	23 (54.8%)
Number of inhabitants			
(a) city	15 (44.1%)	4 (50.0%)	19 (45.2%)
(b) medium-sized town	16 (47.1%)	4 (50.0%)	20 (47.6%)
(c) small town	3 (8.8%)	0 (0%)	3 (7.1%)
Bicycle ownership of child			
(a) yes	34 (100%)	8 (100%)	42 (100%)
(b) no	0 (0%)	0 (0%)	0 (0%)
Child’s ability to cycle			
(a) yes	34 (100%)	8 (100%)	42 (100%)
(b) no	0 (0%)	0 (0%)	0 (0%)
Cycling to school of child (habit)			
(a) yes	22 (66.7%)	5 (71.4%)	27 (67.5%)
(b) no	11 (33.3%)	2 (28.6%)	13 (32.5%)
Cycling to school of child (days/week in M ± SD)	2.6 ± 2.3	3.1 ± 2.2	2.7 ± 2.3
Cycling distance home/school of child (km in M ± SD)	4.3 ± 3.2	5.2 ± 3.2	4.5 ± 3.2
Bicycle ownership			
(a) yes	33 (97.1%)	8 (100%)	41 (97.6%)
(b) no	1 (2.9%)	0 (0%)	1 (2.4%)
Ability to cycle			
(a) yes	34 (100%)	8 (100%)	42 (100%)
(b) no	0 (0%)	0 (0%)	0 (0%)
Employment status			
(a) yes	31 (91.2%)	8 (100%)	39 (92.9%)
(b) no	3 (8.8%)	0 (0%)	3 (7.1%)
Work (days/week in M ± SD)	3.7 ± 1.5	4.9 ± 0.4	3.9 ± 1.4
Cycling to work (habit)			
(a) yes	12 (40.0%)	4 (50.0%)	16 (42.1%)
(b) no	18 (60.0%)	4 (50.0%)	22 (57.9%)
Cycling to work (days/week in M ± SD)	1.3 ± 1.9	1.8 ± 2.2	1.4 ± 1.9
Cycling distance home/work (km in M ± SD)	13.0 ± 14.4	7.9 ± 5.5	11.9 ± 13.2

km = kilometer; M = means; SD = standard deviation.

**Table A4:** Binary Logistic Regressions of Socio-Demographic Characteristics and Child’s Habit of Cycling to School based on the Sample of Parents.

Socio-Demographic Characteristics	Mothers				Mothers and Fathers			
	*p*	OR	95% CI for OR		*p*	OR	95% CI for OR	
			Lower	Upper			Lower	Upper
Gender								
(a) mothers (ref.)								
(b) fathers					0.807	0.8	0.1	4.8
Age (in years)	0.349	1.1	0.9	1.2	0.265	1.1	0.9	1.2
Age of child (in years)	0.103	2.7	0.8	8.8	0.228	1.8	0.7	4.8
Gender of child	0.445	1.8	0.4	7.9	0.308	2	0.5	8
(a) daughter								
(b) son (ref.)								
Educational level/school’s region of child								
(a) high/urban (ref.)								
(b) intermediate/suburban	**0.036**	6.5	1.1	37.5	**0.01**	9.4	1.7	51
Number of inhabitants								
(a) city (ref.)								
(b) medium-sized town	**0.029**	7.4	1.2	45	**0.008**	10.6	1.9	60.2
(c) small town	0.413	3.3	0.2	54.8	0.313	4.3	0.3	70.8
Cycling distance home/school of child (in km)	**0.02**	1.4	1.1	1.8	**0.006**	1.4	1.1	1.8
Employment status								
(a) yes (ref.)								
(b) no	1	1	0.1	12.4	0.974	1	0.1	12.7
Work (in days/week)	0.739	1.1	0.7	1.8	0.764	1.1	0.7	1.7
Cycling to work (habit)								
(a) yes (ref.)								
(b) no	0.103	4.4	0.7	26.7	**0.043**	5.9	1.1	32.9
Cycling to work (in days/week)	0.130	0.7	0.4	1.1	0.063	0.6	0.4	1
Cycling distance home/work (in km)	0.586	1	1	1.1	0.779	1	1	1.1

CI = confidence interval; km = kilometer; OR = odds ratio; *p* = probability value; ref. = reference value.
